# Development and Evaluation of Affective Domain Using Student’s Feedback in Entrepreneurial Massive Open Online Courses

**DOI:** 10.3389/fpsyg.2019.01109

**Published:** 2019-05-24

**Authors:** Wen-Hsiung Wu, Hao-Yun Kao, Sheng-Hsiu Wu, Chun-Wang Wei

**Affiliations:** ^1^ Department of Healthcare Administration and Medical Informatics, Kaohsiung Medical University, Kaohsiung, Taiwan; ^2^ Department of Medical Research, Kaohsiung Medical University Hospital, Kaohsiung, Taiwan

**Keywords:** entrepreneurship education, social entrepreneurship, affective development, MOOCs, content analysis

## Abstract

Entrepreneurship education is a very important issue in the digital age. It aims to enable learners and society to respond to emergent economic and employment challenges. When entrepreneurs struggle to launch and sustain a new venture, the key question usually is not a lack of relevant knowledge, but the necessary fortitude and attitude to face down difficulties and challenges. Thus, entrepreneurs require development in the affective domain. However, most of courses emphasize the cognition and psychomotor functions, but neglect the affective domain. This study attempts to combine entrepreneurial Massive Open Online Courses (MOOCs) and blended curriculum design for affective learning. A total of 32 students participated in a 9-week social entrepreneurship program. Content analysis was used for comparison of the learning performance. The findings suggest that social entrepreneurship courses can be effectively used to help learners achieve learning objectives of different affective levels, but this is a time-intensive process, particularly for higher levels. The affective development of the final level takes longer to achieve; therefore, course designers should adopt a spiral structure which frequently revisits concepts in the last three levels. Moreover, MOOCs are designed for mass usage, and treat all learners uniformly. MOOCs’ course content should be supplemented and adjusted according to specific course goals and student needs.

## Introduction

The digital era is a rapidly changing environment driven by Information and Communication Technologies (ICTs). It not only increases the speed and extent of knowledge throughput in the economy and society, but also creates new opportunities to generate new knowledge more frequently for adaptation to the changing surrounding environment. However, knowledge turnover makes it hard for human beings to control the development and spread of knowledge (Nambisan, 2018). Therefore, how to make good use of the benefits of ICTs in a controlled situation is a very important issue in the digital era.

The European Commission defines entrepreneurship as the ability of individuals to translate ideas into action, including creativity, innovation and risk taking, and the ability to plan and manage projects to achieve goals (Karimi et al., 2016; Palazzeschi et al., 2018). Employees with good entrepreneurship should be able to use emerging technologies to carry out effective activities for value creation. The cultivation of entrepreneurship can help employees better understand their work environment while providing entrepreneurs with the foundation to build new social or business activities to better capture new opportunities (Austin et al., 2006). Thus, entrepreneurship education is an important issue in the digital era.

Entrepreneurship education aims to enable learners and society as a whole to cope with emerging economic and employment challenges through creating an entrepreneurial mind-set and assuming the direct relationship between entrepreneurial intentions, motivation, and attitude (Hytti and O’Gorman, 2004; Haase and Lautenschläger, 2011; Kirby and Ibrahim, 2011; Nabi et al., 2017). Matlay (2008) pointed out that entrepreneurship education will affect attitudes toward entrepreneurship. For example, Walt Disney’s greatest creations are not animated films, not even Disneyland, but their extraordinary ability to delight the audience. If employees lack entrepreneurship, they cannot support the company’s development missions. Entrepreneurship education provides the essential knowledge and skills to increase the number of welleducated entrepreneurs (Matlay, 2008; O’Connor, 2013). Established firms have also reported benefiting indirectly from entrepreneurship education through recruiting better prepared employees (Kuckertz, 2013; Daniel, 2016).

Today, entrepreneurship courses have to cover a wide range of needs for learners of various backgrounds and interests. Bloom’s taxonomy suggests a well-designed course should include the learning objectives in the cognitive, affective, and psychomotor domains (Bloom et al., 1956). However, Sitzmann et al. (2010) pointed out that course design still mostly focuses on knowledge transfer, which emphasizes the cognitive and psychomotor functions, but neglects the affective domain. Moreover, it is difficult to measure achievement of affective goals through traditional evaluation methods. Taber (1989) argued that focusing on cognitive development may leave students unable to adapt to real-world challenges. When entrepreneurs struggle to launch and sustain a new venture, the key challenge usually is not a lack of relevant knowledge, but the necessary fortitude and attitude to face down difficulties and challenges. Thus, entrepreneurs and business managers require development in the affective domain.

Affection and cognition are complementary and cannot be developed independently during the learning process. Kraiger et al. (1993) pointed out that cognitive ability is foundational to affective learning, which is critical to behavioral performance and practical skills. Therefore, affective teaching strategies play an important role in entrepreneurship education (Fodor and Pintea, 2017). Effective teaching in the affective domain can help learners review their value choices, reflect on their value beliefs, revise their value systems, and then create their own approaches for innovation and creativity. However, few studies emphasize the affective domain for entrepreneurship education.

In the digital era, Massive Open Online Courses (MOOCs) offer learning opportunities for updating people’s knowledge of various subjects across the globe (Viswanathan, 2012). Intelligence solutions enabled by new technologies can help learners improve their quality of life and learning performance in the university environment (Coccoli et al., 2014). With the rise of MOOCs, outstanding scholars in individual fields can deliver their knowledge online, thus extending their influence and providing access to students living in peripheral areas. However, different political, economic, social, and educational conditions impact the development of innovation and entrepreneurship in different countries and regions, and the MOOC one-size-fits-all approach presents difficulties in customizing course content (Muñoz-Merino et al., 2015). Previous studies did not provide solutions to design an ideal blended curriculum for improving students’ affective abilities. Thus, this study explores how to balance a non-differentiated curriculum design with regionally specific objectives.

Bloom et al. (1984) argued that affective development is a process of exploring and adapting human interests, attitudes, values, and appreciation. Affective learning outcomes cannot easily be quantified by traditional testing and rather relies on qualitative self-reflection. This study analyzed learning experience reports collected after each class to evaluate whether pre-established teaching objectives had been met. Most previous studies focus exclusively on the assessment of cognitive development; however, this study combines entrepreneurial MOOCs, specific curriculum design, and content analysis for the development and evaluation of the affective domain. Written student feedback and follow-up interviews were the basis for analysis and comparison. The purpose of this study is to investigate how the curriculum design influences the students’ affective development based on Bloom’s taxonomy. The research question is whether the students’ feedback on Bloom’s affective level is different at each stage of the course. The findings may provide useful insight into the development and use of MOOC support for entrepreneurship courses in the digital era.

## Literature Review

### Entrepreneurship Education

Rigg and O’Dwyer (2012) argue there are very different meanings as a curriculum concept between enterprise and entrepreneurship education: enterprise education methods can be “advancing teaching methods” or “including challenging concepts in teaching practice to support and increase problem solving skills” or “improving key employment skills awareness beyond university education.” Even though entrepreneurship education is similar in terms of development and skills development, in many cases, the clear intentions of entrepreneurship and the factors should be considered when choosing employment pathways (Rigg and O’Dwyer, 2012; Gimmon, 2014; Sanchez-Garcia et al., 2018).

The objectives of entrepreneurship education include increasing learner knowledge (i.e., understanding entrepreneurship), improving entrepreneurial abilities and behavior in real-life contexts (i.e., developing an entrepreneurial outlook), and providing a relevant set of skills and competences for establishing new start-ups or managing existing firms (i.e., learning to become an entrepreneur) (Hytti and O’Gorman, 2004; Fretschner and Weber, 2013; O’Connor, 2013). These objectives overlap to some extent, as an increased understanding on the phenomenon of entrepreneurship is likely to influence that learner’s mastery of start-up-related skills or other entrepreneurial competencies (Middleton and Donnellon, 2014; Scott et al., 2016; Thrane et al., 2016).

Jones and Iredale (2010) also argued the key points of entrepreneurship education from the perspectives of different roles, such as policymakers, teachers, lecturers, students, and pupils, focus on start-ups; new enterprise risk planning; initiate, develop, and manage business; develop and operate businesses, advance skills, behaviors, and knowledge needed for self-employment (Jones and Iredale, 2010; Ruskovaara and Pihkala, 2013). On the other hand, entrepreneurship education focuses on developing personal skills, behaviors, attributes, and knowledge needed in a broader range of contexts, with the learner functioning as an employee, consumer, and citizen, with a particular focus on how small businesses work (Thrane et al., 2016). While both domains take similar approaches to developing and improving skills, entrepreneurship education frequently features a clear emphasis on business start-ups and success factors for professional business innovators.

These acknowledgments imply that entrepreneurship education contributes to the development of cognitive, intellectual, and reasoning skills. Participating in entrepreneurship classes, seminar, and internships can positively impact learner affection. Such activity not only develops learner knowledge, attitudes, values, emotions, and skills which contribute to effective self-employment but also teaches them about potential problems which they can avoid (Said, 2014).

### Massive Open Online Course (MOOC)

Self-directed learning can now take place on open online networks which combine communication technologies and extensive resources to create effective online learning environments (Kop, 2011). A massive open online course is an online course which aims to provide unlimited participation and open access *via* the Web. MOOCs represent an innovative, Web-based business model for financing, designing, and delivering educational services (Wulf et al., 2014). Since 2008, MOOCs have been run by a variety of public and private universities, especially in North America. Many academic researchers and practitioners have shown interest in the potential for MOOCs to deliver instruction around the globe on an unprecedented scale, and considerable research attention has focused on developing best practices for the use of these platforms (Liyanagunawardena et al., 2013; Lerís et al., 2017).

MOOCs provide a variety of educational content for those who are willing to learn. Academically, the value of MOOCs to a large number of participants depends on whether MOOCs provide appropriate assistance approaches (Zhuhadar et al., 2015). In addition to traditional course materials such as film lectures, reading and problem sets, many MOOCs offer interactive discussion forums to support community interactions between students, professors, and teaching assistants. MOOC, a recent study extensively in the field of distance education, was first introduced in 2008 and became a popular learning model in 2012 (Saadatdoost et al., 2015). When employees already have entrepreneurial skills, they can overcome the challenges of specific tasks to support the company’s development. Established companies try to recruit more prepared entrepreneurial employees to benefit business operations. Many entrepreneurship courses are offered in MOOC platforms, including Coursera, Udemy, edX, FutureLearn, and Udacity (Spyropoulou et al., 2014). These courses explore entrepreneurship-related issues from diverse perspectives, such as innovation and creativity, design thinking, product design, entrepreneurial mind-set, business strategies, financial planning, social enterprise, and so on. These online courses provide good guidance to help those who attend classes succeed in entrepreneurship.

### Affective Domain Objectives

Bloom’s et al. (1956) taxonomy divides educational objectives into three overlapping “domains”: cognitive (knowledge), affective (attitude), and psychomotor (skills). The model supports effective student learning by helping teachers determine the appropriate teaching strategies to be used (Bloom et al., 1956, 1984; Savickienë, 2010; Testa et al., 2018). The Taxonomy of the Affective Domain contains five levels, from lowest to highest: receiving, responding, valuing, organization, and characterization (Krathwohl et al., 1964; Anderson et al., 2001). This taxonomy was applied to written self-evaluations to assess changes in affective learning. Each level is described as follows (Krathwohl et al., 1964; Anderson et al., 2001):

*Receiving*: Awareness of the need and willingness to hear selected attention, e.g., listening respectfully to others, listening for and remembering names of newly introduced people. Keywords for content analysis include acknowledge, ask, attentive, courteous, dutiful, follows, gives, listens, and understands. When students present these keywords in their written feedback and the meaning of the sentence conforms to the concept of this level, it will be encoded as belonging to this level.

*Responding*: Actively participate in learning, including responding to various appearances. Learning outcomes may emphasize compliance in response, willingness to respond, or satisfaction (motivation) in response. Examples include participation in class discussions, presentations, questions to improve understanding, and compliance with safety rules. Keywords at this level include answers, assistants, assists, compliance, compliance, discussions, greetings, help, tags, shows, gifts, and narration.

*Valuing*: It is defined as the ability to judge the worth or value of something, including specific objects, phenomena, behaviors or information, and to express it clearly from simple acceptance to a more complex state of commitment. When a learner internalizes a particular set of values, these value beliefs can usually be expressed by explicit and identifiable behaviors. Examples include expressing convictions about the democratic process, being sensitive to individual and cultural differences (i.e., focusing on diversity), addressing value conflicts, proposing social improvement plans and fulfilling commitments, and informing management of concerns. Keywords of this level include appreciates, cherish, treasure, demonstrates, initiates, invites, joins, justifies, proposes, respect, and shares.

*Organization*: It is defined as comparing and classifying values, resolving conflicts between them, and creating a unique value system with a primary focus on comparison, relevance, and integrated values. Case in point includes recognizing the need for an equilibrium between freedom and responsibility, explaining the importance of system planning in solving problems, accepting ethical standards, creating life plans that suit their abilities, interests, and beliefs, effectively prioritizing time to meet organizations, family, and self-needs. Keywords of this level include compares, relates, and synthesizes.

*Characterization*: It is defined as the establishment of a value system that controls learner behavior, which is universal, consistent, predictable, and the most important feature of learners. Teaching objectives involve individual, social, and emotional patterns that learners adjust. For example, being able to work independently, collaborate in group activities, use objective methods to solve problems, practice professional ethics, modify beliefs and change behavior based on new evidence, and value people beyond superficial features. Keywords of this level include acts, discriminates, displays, influences, modifies, performs, qualifies, questions, revises, serves, solves, and verifies.

Entrepreneurship courses differ from general management courses in that the former aim to not only provide knowledge, but also to prepare students to launch a business. Entrepreneurship education is also more action oriented than general education, which seeks to cultivate professionalism. One of the main goals of the social studies curriculum is to promote the emotional development of students, including improving their interest in learning, positive attitudes, and local cultural identity. However, it is difficult to construct or change the attitudes of students. Affection-related teaching objectives are not as clearly defined as those for cognitive or action-related skills (Hwang and Chang, 2016). To effectively achieve learning objectives, entrepreneurship courses must properly manage student interest in learning over time, while promoting good ethics. To help students develop positive value systems and attitudes, entrepreneurship courses not only focus on cognitive and psychomotor development, but consider the influences of affective domain (Jagger, 2013).

## Materials and Methods

### Course Design

Recently, an emphasis of entrepreneurship activity has emerged to generate social benefits. So-called social entrepreneurship uses innovative approaches to address problems in the domains of education, environmental protection, fair trade, health and human rights, and is widely regarded as an important building block for sustainable national development (Mair and Marti, 2006). Peter Ferdinand Drucker has suggested that social entrepreneurship may eventually become more important than for-profit entrepreneurship (Mair and Noboa, 2006; Ruskin et al., 2016). Therefore, a social entrepreneurship course from Coursera was selected for this study.

A 9-week blended course was designed to integrate affective learning into an international MOOC curriculum. The course title was Introduction to Social Innovation and Entrepreneurship, a third-grade course in the Department of Healthcare Administration and Medical Informatics at a medical university in Taiwan. This introductory course provides students with an understanding of the areas of social innovation and social entrepreneurship and introduces students to several useful “frameworks” to understand the field and apply it to follow-up courses in social innovation and social entrepreneurship. The blended approach is that after each week’s MOOC session, the instructor conducted affective domain learning activities (primarily through case studies and group discussions) in local classrooms to achieve affective targets for entrepreneurial education. Canaleta et al. (2014) mentioned that active learning methodologies enhance the development of the competences of students and provide a better evaluation of outcomes. The learning activities of this study encouraged students to participate directly and actively in the learning process. The instructional design is summarized in Table 1. A total of 32 students (12 male and 20 female, ranging in age from 21 to 24 years) participated in the course. Oral informed consent was obtained from all participants. Each week, the learners provided written feedback on slips of paper after each class and these comments served as the basis for further content analysis. These open-ended questions include: What did you learn in this course? What difficulties have you encountered? Evaluate the strengths and weaknesses of the course content? What is your our own opinion on this issue and other ideas.

**Table 1:** Entrepreneurship course objectives.

Week	Affective level	Course objectives
1	Receiving and responding	Introduction to “social entrepreneurship”
2	Receiving and responding	Defines social entrepreneurship through a case study of Bangladesh’s Grameen Bank. Students are assigned to develop a proposal within the CBS Entrepreneurship Platform to attract external funding
3	Receiving and responding	Discuss the characteristics needed for successful social entrepreneurship
4	Valuing and organization	Identifying and developing opportunities: identifying hidden ones, creating new ones, eliminating the need for one and creating demand for antagonistic assets
5	Valuing and organization	Distinguishing business models for specific businesses in terms of scale model, role model, organism, recipes
6	Valuing and organization	Applying the “business model canvas” concept to the real businesses
7	Characterization	Discussing and developing business proposals
8	Characterization	Optimizing organizational structures using examples from CIC and L3C. Identifying the pros and cons of different organization types
9	Characterization	Attracting external funding. Students share their experience of developing effective business plans for raising funds

### Content Analysis

In order to understand the real impact of the blended teaching strategy on learners, the field research method was applied in this study. Field research is a non-experimental scientific inquiry to explore the relationship and interaction between educational, psychological, and social variables in real situations (Burgess, 2002). The research method does not have any experimental manipulation or random sampling of research subjects or distribution groups. Everything was done in a natural situation. After collecting student feedback, content analysis was conducted using individual sentences as the unit of analysis to identify consistent units of meaning (e.g., themes or ideas) in a message. Berelson (1952) defined content analysis as “a research technique for the objective, systematic and quantitative description of the manifest content of communication.” We followed Berrellson’s (1952) opinion to analyze students’ feedback into quantitative charts systematically and then explained the findings. To ensure inter-rater reliability, we created two coding teams, each consisting of a researcher and a research assistant with backgrounds in entrepreneurship education and educational technology. Bloom’s classification guide was used as a standard for content analysis. A coding manual detailing coding instructions and standardized coding worksheets was prepared and distributed to both teams. The coders read the student’s weekly written feedback. If the text is related to the affective domain, it is classified at a certain affective level. Their coding tasks were processed based on the levels of affective domain, such as receiving, responding, valuing, organization, and characterization as proposed by Bloom et al. (1956).

Krathwohl et al. (1964) defined action verbs appropriate for each level of Bloom’s taxonomy. The action verbs can be used to indicate a clearly observable and measurable action. We only counted sentences with the action verbs in the affective domain. Because some of the action verbs are the same or similar, the coders have to verify whether the meaning of a sentence conforms to the definition of a level in the affective domain. The action verbs for each level of affective domain are accept, attend, develop, recognize for Receiving; complete, comply, cooperate, discuss, examine, obey for Responding; accept, defend, devote, pursue, seek for Valuing; codify, discriminate, display, order, organize, systematize, weigh for Organization; and internalize, verify for Characterization (Krathwohl et al., 1964).

The actual coding process was preceded by training sessions and discussion of the coding instructions. Text coding is inherently subjective, too often leaving the results open to the coder’s personal preferences or biases. This potential for bias makes it essential to check results between individuals and teams. Individual discrepancies were discussed to reach a group consensus. To improve scoring consistency, the coders conferred and compared results openly at the end of the first unit, and constant communication between individuals and teams was encouraged to ensure solutions and conclusions were shared throughout all groups. The actual coding process commenced after the second discussion to ensure each coder clearly understood the requirements.

Thirty-two students produced 288 course feedbacks in 9 weeks. These feedbacks were split into 2,938 sentences analyzed. Each person contributed about 10 sentences per week. There were 714 sentences related to the affective domain, accounting for 24.3% of the total sentences. Once coding was completed, coders exchanged their results with each other to perform a pair inter-rater reliability check and reliability indexing. Inter-rater reliability testing found agreement of 84.76% with a Kappa of 0.76.

### Follow-Up Interviews

To understand the retention rate of students in the affective domain, follow-up interviews were conducted to gain an understanding of participant feedback about the affective domain 2 weeks after the end of the course. Interviews were conducted with eight students (three male and five female) who participated in the course. The interviews consisted of open-ended questions designed to evaluate the impact of course, content, and platform design on affective development. Later, follow-up individual interviews lasting 40 min each were conducted to validate participant response to these questions. More importantly, the results of interviews were applied to corroborate the previous analysis of other data sources (i.e., content analysis), to link and compare with the results of content analysis. A total of 201 sentences from eight students were analyzed. Each person contributed about 25 sentences to the interview. There were 71 sentences related to the affective domain, accounting for 30.3% of the total sentences.

Two researchers independently analyzed the data and established the inter-rater agreement from the interview results, producing an agreement rate of 92%. Each participant was treated as a separate case. Data from each interview were used to generate a summary of the participant’s views on and conceptions of the entrepreneurship course. These summaries were then reviewed and discussed by the researchers to reach a consensus on the affective domain about the entrepreneurship course assisted with MOOCs. These results were then compared against those generated from content analysis.

## Results

Affective development is time-consuming and requires specially designed teaching methods. In addition, its learning outcomes are difficult to evaluate (Pascarella, 1985). As a result, most courses emphasize cognitive and psychomotor development, and generally neglect the affective domain. In the digital era, the development of affective abilities can help people adapt to the rapidly changing environment. MOOC-based courses allow for nearly universal access to instructional content delivered over the Internet. However, the content may not fit the needs of regional courses. The entrepreneurship course of this study is supplemented with instructional content intended to emphasize affective development and achieve a balance with cognitive development.

Table 1 shows the affective teaching objectives for the 9-week course. The first 3 weeks focused on receiving and responding, while helping students develop a better understanding of social issues and social entrepreneurship operations. Weeks 4–6 emphasized valuing and organization, encouraging students to develop their own definitions of social enterprises and to distinguish social enterprise characteristics in different national contexts. The last 3 weeks of the course gradually shifted the focus to characterization, encouraging students to make ethical judgments and to evaluate feasible solutions for different social problems. As the course progressed, learners engaged in activities designed to provide exposure to successive affective domain levels, with considerable overlapping between stages. At the conclusion of the course, students were expected to be able to sum up their acquired knowledge for establishing social enterprises and contributing to the solution of social issues.

**Figure 1:**
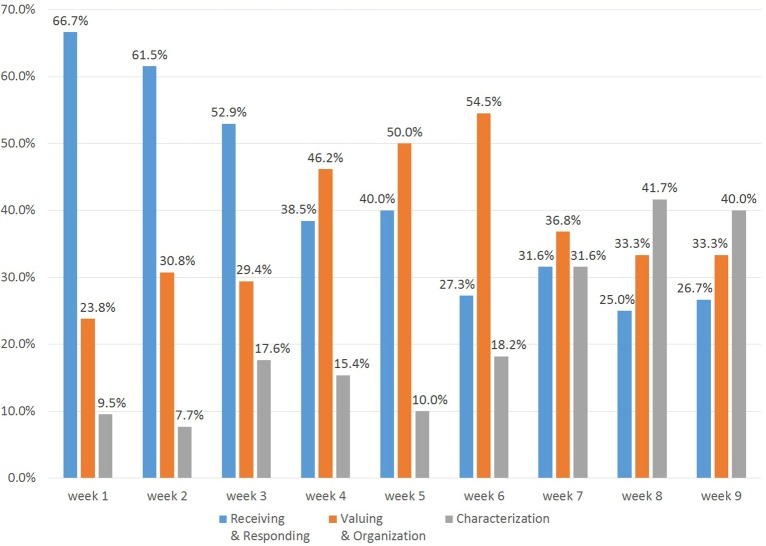
Variation of affective goals.

After each class session, students were asked to provide written feedback, and these comments were used as data for affective development analysis. This study only focuses on the feedback content in the affective domain. To avoid bias introduced by the small amount of feedback at each level, the five levels of the affective domain were simplified into three categories: low (receiving and responding), middle (valuing and organization) and high (characterization). Figure 1 shows the variation between categories and a timeline of affective goals. It was found that course design can significantly influence affective domain development.

**Figure 2:**
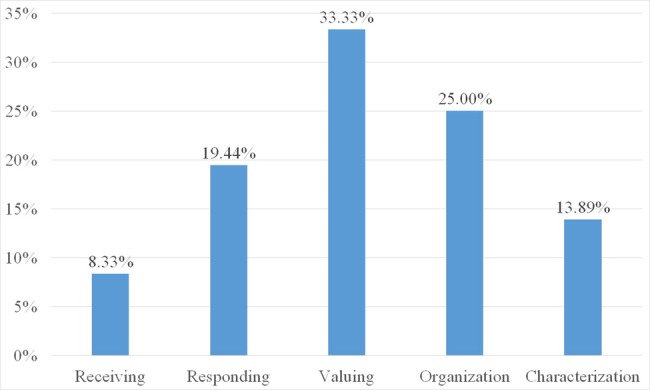
The retention status after the end of the course.

Two weeks after the end of the course, the students were invited to reflect on the whole course to assess the retention of affective learning, as shown in Figure 2. The results show that the frequency related to middle level of affective domain in students’ written feedback was higher than the frequency related to low and high levels.

## Discussion

In the first stage, low-level affective concepts are considerably more developed than the middle and high levels. This early stage emphasizes receiving and responding skills, with the instructor guiding students in understanding the basic concepts, opportunities, challenges, and resources of the social entrepreneurship, which helped students understand and pay attention to relevant issues, and then improve their entrepreneurial motivation. Before the student’s motivation is aroused, they may begin with passive attitudes toward the contents and the teacher’s concepts. Further guidance should encourage students to take the initiative rather than just participate passively.

The second stage shifted the focus to mid-level affective skills; therefore, the middle level feedback is higher than that of low level, prompting students to consider the importance and usefulness of entrepreneurial values and adopt them. Different entrepreneurial values found different degrees of acceptance in terms of cognition and affection. The course design encourages students to evaluate and select entrepreneurial values on their own, and to gradually accept and internalize various aspects of entrepreneurial knowledge. After the establishment of the value assessment process, students will consider different values and address conflicts from the viewpoint of social enterprise to establish an internally consistent value system. The value organization emphasizes the comparison, relation, and integration of various values.

Later stage development enhances high-level skills, which contribute to the development of characterization, and these positive changes in personal value systems are internalized in learners’ thoughts and characters.

The follow-up interview implies that the students’ affective development was still in the valuing and organization levels, having progressed beyond the receiving and responding levels, but they had not yet internalized the characterization level. The affective development of the final level takes longer to achieve, thus course designers should adopt a spiral structure which frequently revisits concepts in the last three levels. Moreover, the high affective level may be retained for a long duration if classroom-based instruction can be supplemented by hands-on experience or field practice opportunities. The arrangement of programs and teaching methods, which followed the principle of learning by doing, can not only enable students to balance theory and practice, but also support students in creating social enterprises (Wu et al., 2013; Antonaci et al., 2015).

## Conclusion

The achievement of affective teaching goals is an important task for entrepreneurship education in the digital era. Guiding students to reflect on and revise their values and social beliefs is a time- and labor-intensive endeavor, requiring considerable effort for effective evaluation. Weekly feedback and interaction between teachers and students can enhance student enthusiasm for entrepreneurship.

Our findings suggest that social entrepreneurship courses with blended approach can be effectively used to help learners achieve different levels of affective domain teaching objectives, but this is a time-intensive process, particularly for higher levels. Affective development at the receiving and responding levels can be reached in 3 weeks, while 5 weeks should be allocated for valuing and organization, and at least 7 weeks for the characterization level. Higher levels of development take longer to achieve; therefore, course designers should adopt a spiral structure which frequently revisits concepts in the last three levels. Moreover, MOOCs are designed for mass usage and treat all learners uniformly. MOOCs’ course content should be supplemented and adjusted according to specific course goals and student needs.

The main limitation of this study is the use of field research. Since the participants of this study are students of a course, researchers can invite various types of subjects and use experimental methods to explore the impact of various teaching strategies on the students’ affective development. In addition, this study presents the learners’ affective feedback in a quantitative manner. In the future, researchers can develop a causal model to explore the factors that influence the affective development of learners.

## Ethics Statement

Oral informed consent was obtained from all participants. For the reported study, no ethics approval was required according to the guidelines of Kaohsiung Medical University or Ministry of Health and Welfare of Taiwan.

## Author Contributions

W-HW, H-YK, and C-WW contributed to the supervision of the project, design of the research, organization of experiment conduction, data analysis and interpretation, writing and revision of the article. S-HW contributed to the organization of experiment conduction, data analysis and interpretation, and writing of the article.

### Conflict of Interest Statement

The authors declare that the research was conducted in the absence of any commercial or financial relationships that could be construed as a potential conflict of interest.
